# A Porcine Epidemic Diarrhea Virus Isolated from a Sow Farm Vaccinated with CV777 Strain in Yinchuan, China: Characterization, Antigenicity, and Pathogenicity

**DOI:** 10.1155/2023/7082352

**Published:** 2023-03-07

**Authors:** Jianlin Lei, Yongqiang Miao, Zhao Guan, Hui Chen, Chaohui Xiang, Hangqi Lu, Yuan Fang, Yu Han, Ruochen Hu, Kejia Lu, Zhengwu Chang, Xinglong Wang, Shuxia Zhang, Haijin Liu, Zengqi Yang

**Affiliations:** ^1^College of Veterinary Medicine, Northwest A&F University, Shaanxi, Yangling, China; ^2^College of Agriculture and Forestry Science and Technology, Longdong University, Qingyang, China

## Abstract

Porcine epidemic diarrhea virus (PEDV) is a porcine enteric coronavirus globally, causing serious economic losses to the global pig industry since 2010. Here, a PEDV CH/Yinchuan/2021 strain was isolated in a CV777-vaccinated sow farm which experienced a large-scale PEDV invasion in Yinchuan, China, in 2021. Our results demonstrated that the CH/Yinchuan/2021 isolate could efficiently propagate in Vero cells, and its proliferation ability was weaker than that of CV777 at 10 passages (P10). Phylogenetic analysis of the S gene revealed that CH/Yinchuan/2021 was clustered into subgroup GIIa, forming an independent branch with 2020-2021 isolates in China. Moreover, GII was obviously allocated into four clades, showing regional and temporal differences in PEDV global isolates. Notably, CH/Yinchuan/2021 was analyzed as a recombinant originated from an American isolate and a Chinese isolate, with a big recombinant region spanning ORF1a and S1. Importantly, we found that CH/Yinchuan/2021 harbored multiple mutations relative to CV777 in neutralizing epitopes (S1^0^, S1^A^, COE, and SS6). Homology modelling showed that these amino acid differences in S protein occur on the surface of its structure, especially the insertion and deletion of multiple consecutive residues at the S1^0^ epitope. In addition, cross-neutralization analysis confirmed that the differences in the S protein of CH/Yinchuan/2021 changed its antigenicity compared with the CV777 strain, resulting in a different neutralization profile. Animal pathogenicity test showed that CH/Yinchuan/2021 caused PEDV-typified symptoms and 100% mortality in 3-day-old piglets. These data will provide valuable information to understand the epidemiology, molecular characteristics, evolution, and antigenicity of PEDV circulating in China.

## 1. Introduction

Porcine epidemic diarrhea virus (PEDV) is an important enteropathogenic agent, which causes porcine epidemic diarrhea (PED) characterized by watery fetid diarrhea, vomiting, dehydration, and marked emaciation affecting all ages, with 100% morbidity and 80–100% mortality in neonatal piglets [1–3]. Originally identified in the UK in 1971, it had been spread through European countries, with sporadic outbreaks in the late 20th century [4, 5]. A similar PEDV-induced diarrhea was first reported in China in 1973, and PEDV was identified in 1984. From 1984 to early 2010, the PEDV-caused epidemic was widespread in different provinces of China, with mostly sporadic or local epidemics [6]. A large-scale PED outbreak occurred in southern China since 2010, infecting pigs of different ages, and the mortality rate of newborn piglets reached 100%, which rapidly spread throughout the country [7, 8]. Studies have confirmed that the novel PEDV variants with high pathogenicity are the main cause for these outbreaks in China [8–10]. In 2013, PEDV was first reported in the United States, followed by the emergence of a highly pathogenic strain of PEDV that rapidly infected pig populations [11, 12], it then rapidly spread through the pig industry in European, North America, and Asia countries, resulting in huge economic threats to the pig industry globally [1, 13–15]. In recent years, although PEDV has been basically controlled in China through improving biosafety measures and extensive application of traditional vaccine (inactivated or attenuated CV777) and new vaccine (inactivated PEDV variant AJ1102), PEDV is still circulating in China due to high genetic heterogeneity [16, 17].

PEDV, a positive sense and single-stranded RNA virus, belongs to the family *Coronaviridae*, genus *Alphacoronavirus* [18]. The genome is approximately 28 kb, containing two replicase polyproteins: open reading frames (ORFs) 1a and ORF1b, four structural proteins: spike (S), envelope (E), membrane (M), and nucleocapsid (N), and one accessory protein encoded by ORF3 [15]. The S protein plays a key role in viral entry, which can be divided into S1 and S2 subunits [19]. S1 is responsible for receptor binding, containing the *N*-terminal domain (NTD) and C-terminal domain (CTD) [20]. The S2 is involved in triggering virus-cell membrane fusion during virus entry [21]. The S protein is considered to be a primary protein in inducing neutralizing antibodies, which contains six neutralizing epitope regions: S1^0^ (aa 19–220) [22], S1^A^ (aa 435–485) [23], COE (aa 499–638) [24], SS2 (aa 748–755), SS6 (aa 764–771) [25], and 2C10 (aa 1368–1374) [26]. In addition, the PEDV S gene is prone to mutation and exhibits a high genetic diversity, which is continually used to assess the genetic diversity and virulence of the strains circulating in the field [27, 28].

Based on phylogenetic analysis of the S gene, PEDVs are divided into two obvious genotypes: I (GI) and II (GII) and further clustered into five subgroups: GIa, GIb, GIc, GIIa, and GIIb [8, 17]. Depending on the S gene and pathogenicity, PEDVs can also be classified into the highly pathogenic non-S-INDEL genotype, and milder virulence S-INDEL genotype with insertions and deletions of the NTD of the S gene [4, 29]. GII and GIc genotypes correspond to the non-S-INDEL and S-INDEL genotypes, respectively, [17]. At present, GII is the main epidemic strain circulating in China, posing a serious challenge to the pig industry [8]. It is well reported that the pathogenicity of the PEDV S-INDEL strain is milder than that of non-S-INDEL highly pathogenic strains, and both variants have been documented in Asian and North American countries [17, 30]. In the present study, a PEDV strain CH/Yinchuan/2021, was isolated in a sow farm vaccinated with the CV777 strain in Yinchuan, China. Furthermore, we analyzed the growth characteristics, evolution, recombination, and antigenicity of CH/Yinchuan/2021 and evaluated its pathogenicity in piglets. These data are useful for understanding the prevalence, evolution, molecular characteristics, and immunogenicity of PEDV circulating in China.

## 2. Materials and Methods

### 2.1. Sample Collection and Testing

In July 2021, an outbreak of severe diarrhea and death of piglets occurred in 7 adjacent sow farms in Yinchuan, Ningxia Hui Autonomous Region of China. The cases were characterized by yellow and watery diarrhea, dehydration, vomiting, and emaciation, with 60–100% morbidity and 80–100% mortality in neonatal piglets, while the affected sows are characterized by anorexia, vomiting, and diarrhea but recovered within 1 week. In 7 affected sow farms, all sows were vaccinated with the PEDV CV777 attenuated vaccine and PEDV-TGEV live attenuated vaccine. Nine intestinal contents and 47 fecal samples were collected from dead or live piglets, and 16 serum samples were collected from pre- and postpartum sows. Eight PEDV-negative serum samples were collected from sows in a nonimmunized household. Intestinal contents and fecal samples were diluted using Dulbecco's modified Eagle's medium (DMEM, Gibco, USA) to be 10% (wt/vol) suspensions. The suspension was then centrifuged at 5,000 ×g for 30 min at 4°C, filtered by a 0.22 *μ*m pore-size syringe filter (Biosharp, Beijing, China), and the filtrate was used as inoculum for virus isolation.

To detect the PEDV in the collected samples, virus RNA was extracted from the samples (feces and intestinal contents) using RNAiso reagent (TaKaRa, Dalian, China) according to the manufacturer protocols. Complementary DNA (cDNA) was synthesized employing a Star Script II First-strand cDNA synthesis kit II (Genstar, China) according to the manufacturing instructions. Primers for RT-PCR were designed with Primer Premier 5.0 software targeting a 668-bp fragment based on conserved regions of the PEDV M gene (PEDV-M-F: 5′-ACG GTT CTA TTC CCG TTG ATG-3′; PEDV-M-R: 5′-TAA ATG AAG CAC TTT CTC ACT ATC-3′). PCR conditions were as follows: denaturation at 95°C for 2 min, 35 cycles of 95°C for 30 s, 55°C for 30 s, and 72°C for 45 s, followed by a final extension at 72°C for 5 min. The PCR products were analyzed by agarose gel electrophoresis. In addition, the other three diarrhoea-related enteric viruses including porcine delta coronavirus (PDCoV), porcine rotavirus (PoRV), and transmissible gastroenteritis virus (TGEV) were detected using specific RT-PCR according to previously reported methods [31, 32].

### 2.2. Virus Isolation, Propagation, and Purification

PEDV isolation was used by Vero cells (ATCC, CCL-81) as previously described with some modifications [33]. Briefly, Vero cells were cultured at 37°C in a 5% CO_2_ incubator and maintained in DMEM (Gibco, USA) supplemented with 10% heat-inactivated fetal bovine serum (FBS, Gibco, USA), 10 unit/ml penicillin and 10 *μ*g/ml streptomycin. Before infection, the inoculum was supplemented with the maintenance media containing 10 *μ*g/ml trypsin (Gibco, USA) and antibiotics and incubated in an incubator for 1 h. Confluent Vero cells in 6-well plates were washed three times with the maintenance media and then incubated with 500 *μ*l inoculum. After a 2 h incubation at 37°C with 5% CO_2_, the inoculum was removed and 2 ml maintenance media was added to each well. The inoculated cells were cultured at 37°C in a 5% CO_2_ incubator and checked daily for cytopathic effects (CPE). Upon the development of 80% CPE, inoculated cells were conducted to three times of freezing and thawing to release an intracellular virus into the medium. The culture supernatants were centrifuged at 1,500 ×g for 10 min at 4°C, and then the clarified supernatants were collected for saved at −80°C. If no CPE is observed after 7 days, the inoculated cells were subjected to freeze-thaw once, and the supernatants were collected by centrifugation and inoculated onto freshly prepared Vero cells for serial passages. The inoculated cells of each passage were identified by the immunofluorescence assay (IFA) and RT-PCR. If CPE, IFA, and RT-PCR were negative after 5 passages, the virus was considered to have failed to be isolated successfully. If the above-given three methods were all positive, the virus was successfully isolated, and purified by plaque purification as previously described [34]. PEDV CV777 strain was preserved in our laboratory. The PEDV isolated in this study was defined as CH/Yinchuan/2021.

### 2.3. Virus Titration and Growth Curve PEDV

Vero cells were seeded in 100 *μ*l medium at a density of 3 × 10^5^ cells/well into 96-well plates (Corning, Lowell, MA) and cultured for 24 h at 37°C in a 5% CO_2_ incubator. Ten-fold serial dilutions of the PEDV CV777 and CH/Yinchuan/2021 strains were added to the wells with 100 *μ*l of diluted virus per well. After 3 days of inoculation, the plates were performed to IFA staining and the PEDV CV777 and CH/Yinchuan/2021 strains titers were calculated as the 50% tissue culture infective dose (TCID_50_)/ml based on the bibliography of Reed and Muench method.

The growth curve of PEDV CV777 and CH/Yinchuan/2021 strains in Vero cells was determined according to TCID_50_. The CV777 and CH/Yinchuan/2021 were inoculated onto Vero cells in 6-well plates at 0.1 multiplicity of infection (MOI). After a 2-h adsorption at 37°C with 5% CO_2_, the cells were maintained with 2 ml maintenance media and harvested for virus titration every 8 h postinfection (hpi). The supernatants were collected after centrifugation, and the TCID_50_/ml was determined by the Reed and Muench method. Virus titration was performed with triplicates at different time points.

### 2.4. IFA

To identify PEDV propagation in each passage, Vero cells were infected with inoculum or PEDV CH/Yinchuan/2021 (0.1 MOI). The cells were fixed with 4% paraformaldehyde at 24 hpi for 30 min at room temperature (RT), washed three times with phosphate buffered saline (PBS), and blocked with PBS containing 1% bovine serum albumin (BSA) at RT for 30 min. After washing three times with PBS, cells were blocked with 5% skim milk for 1 h and then incubated with PEDV-N protein polyclonal antibody (1 : 2000; prepared in our laboratory) for 2 h. The cells were incubated for 1 h at RT with a goat antimouse secondary antibody conjugated to fluorescein isothiocyanate (FITC) (Abcam, Shanghai, China) after washing three times with PBS, and counterstained with 4′,6-diamidino-2-phenylindole (DAPI; Sigma; 1 : 1000). The green signal was visualized employing a fluorescence microscope (IX73; OLYMPUS, Tokyo, Japan).

### 2.5. Western Blot Analysis

To investigate the expression of PEDV N protein, Vero cells were infected with PEDV CH/Yinchuan/2021 at 0.1 MOI, washed with PBS at 24 hpi, and then lysed using RIPA Lysis buffer (Solarbio Life Sciences, Beijing, China). The lysates were mixed with 5 × sodium dodecyl sulfate polyacrylamide gel electrophoresis (SDS-PAGE) loading buffer, boiling for 5 min, separated by SDS-PAGE with 10% polyacrylamide gels and transferred onto a nitrocellulose blotting membrane. Next, the membranes were blocked in 10% skim milk in Tris-buffered saline containing Tween-20 (TBST) overnight at 4°C and then incubated with PEDV-N protein polyclonal antibody (1 : 3000) at 37°C for 2 h. The membrane was incubated with the HRP-conjugated goat antimouse IgG (1 : 5000; Invitrogen, Carlsbad, CA, USA) at 37°C for 1 h after washing three times with TBST. The protein bands were visualized with a chemiluminescence imager (MiniChemi610; Sagecreation, Beijing, China). The protein load was normalized to the Tubulin (1 : 3000; Sungene Biotech, Tianjin Binhai New Area, China).

### 2.6. Full-Length S Gene and Whole Genome Sequencing

To further identify PEDV in 7 affected sow farms, all PEDV-positive samples (feces and intestinal contents) were selected for S gene sequencing using the traditional Sanger method. To obtain the S gene sequences, virus RNA was extracted from the samples (feces and intestinal contents) using RNAiso reagent (TaKaRa, Dalian, China), and cDNA was synthesized using a Star Script II First-strand cDNA synthesis kit II (Genstar, China) according to the manufacturing instructions; the S gene of PEDV was amplified and sequenced according to the previously described protocol [13]. Sequences were assembled and analyzed using Lasergene DNASTAR™ software (DNASTAR, Inc. USA, Wisconsin, Madison). PEDV CH/Yinchuan/2021 whole gene sequencing was conducted at Shanghai Tanpu Biotechnology Co., Ltd (Shanghai, China), using next-generation sequencing (NGS) technology. RNA was extracted from PEDV CH/Yinchuan/2021 (P4) infected cell lysate using RNAiso reagent (TaKaRa, Dalian, China) and prepared for next-generation sequencing. Briefly, reverse transcription used random hexamers. Subsequent DNase treatment and cleanup was followed by second-strand synthesis before library preparation using Nextera XT reagents (Illumina) and sequencing on the NovaSeq 6000 (Illumina). Sequence data were assembled and analyzed as described previously [35].

### 2.7. Phylogenetic and Recombinant Analysis

The sequences of reference PEDV strains are downloaded from the GenBank (Table S1), and the nucleotide sequences of the S gene and full-length genome of PEDV were aligned employing Clustal W in Lasergene MegAlign (DNASTAR, USA) and MEGA software version 7 software. The phylogenetic tree was constructed utilizing the neighbour joining method based on the maximum composite likelihood method and bootstrap methods estimated for 1,000 replications. The phylogenetic tree was visualized using the Interactive Tree Of Life (iTOL) software (http://itol.embl.de/). The potential recombinants of CH/Yinchuan/2021 were analyzed employing the Recombination Detection Program (RDP) (v.4.101) with RDP, GENECONV, BootScan, MaxChi, Chimaera, SiScan, and 3Seq in default mode. Putative recombination is considered significant only if it is supported by at least five of the above recombination detection algorithms (*p*-value <10^−6^).

### 2.8. Protein Structural Analysis

The PEDV S aa sequence of CV777 (GenBank No. AAK38656.1) was retrieved from the GenBank database. The monomeric PEDV S was modelled using the SWISS-MODEL server (https://swissmodel.expasy.org/) with the S protein of GIIb PEDV (PDB ID: 7w6m) [36] as a template. The best homology model was selected based on the GMQE (global model quality estimation). Comparisons and annotations of structures were employed with PyMOL (The PyMOL Molecular Graphics System, Version 1.7.4 Schrödinger, LLC).

### 2.9. Construction of Plasmids

The S1 and S2 genes of PEDV CV777 and CH/Yinchuan/2021 strains were amplified by two pairs of primers (Table S2), and the S1 and S2 fragments were fused into the full-length S gene sequence using overlap extension PCR. The purified PCR products were then cloned into the expression vector pET-32a (Novagen, Madison, USA) using *EcoR I* and *Xho I* cloning sites. After culturing the positive clones, the plasmids were identified using double digestion identification and PCR. The recombinant plasmid with the correct sequence was named pET-32a-CV777-S and pET-32a-CH/Yinchuan/2021-S.

### 2.10. S Protein Expression and Purification

The constructed recombinant plasmids were transformed into *E. coli* BL21 (DE3) for the expression of His-tagged PEDV S protein. The transformed BL21 was kept at 37°C in LB medium supplemented with 50 *μ*g/ml ampicillin. Protein expression was induced by adding isopropyl-*β*-D-thiogalactopyranoside (IPTG) (Sigma-Aldrich) at a final concentration of 1 mM for 6 h at 30°C. After induction by IPTG, bacteria were collected by centrifugation at 4°C and disrupted by ultrasonication. The expressed protein was analyzed by SDS-PAGE (6% polyacrylamide gels) with Coomassie brilliant blue R250 (Solarbio, Beijing, China) and confirmed by Western blotting using anti-His mouse monoclonal antibody (MAb) (1 : 3000; Cell Signaling Technology, Inc., USA). The pET-32a empty vector was used as a control for parallel analysis. The recombinant proteins were purified using the His-tag Protein Purification Kit (Beyotime Biotech Inc, Shanghai, China) according to the standard protocol.

### 2.11. Immunization in Mice

The PEDV CV777 and CH/Yinchuan/2021 strains with the same virus titer (10^6^ TCID_50_/ml) were inactivated with 4% formaldehyde solution at 37°C. The inactivated viruses were refrigerated at 2°C to 4°C for further use. Forty, 6-week-old female BALB/c mice were obtained from Chengdu Dossy Experimental animals Co., Ltd, Chengdu, China, and randomly divided into five groups (*n* = 8). After one week of acclimation, mice were immunized subcutaneously with 200 *μ*l of inactivated PEDV CV777, CH/Yinchuan/2021 or PBS (control group), and 50 *μ*g/mouse of purified CV777-S or CH/Yinchuan/2021-S recombinant protein, respectively. Mice were inoculated two times (inactivated PEDV) at 0 and 14 days (S protein was immunized four times at 0, 14, 21, and 28 days) with the same dose and route, respectively. The inactivated PEDV and purified proteins are mixed with Freund's adjuvant (Sigma–Aldrich) in the same volume prior to injection. The first immunization was accompanied with a complete Freund's adjuvant, and the subsequent booster immunizations were accompanied with an incomplete Freund's adjuvant. At 28 days (S protein at 35 days), all mice were sacrificed according to the approved ethical protocol, and serum samples were collected for further analysis.

### 2.12. Virus Neutralization Assay

PEDV-specific neutralizing antibodies in sera collected from sows and mice were determined by a virus neutralization test (VNT) as described previously [37] with some modifications. Briefly, the collected serum samples were inactivated at 56°C for 30 min and serially diluted two-folds and mixed with an equal volume of 200 TCID_50_ of PEDV (CV777 or CH/Yinchuan/2021 strain) in each well. After a 1 h incubation at 37°C, 100 *μ*l of the mixture was transferred to Vero cell monolayers and incubated at 37°C for 1 h. Then, the inocula were discarded and Vero cells were washed three times with DMEM. Thereafter, 100 *μ*l DMEM containing 10 *μ*g/ml trypsin (Gibco, USA) was added to the cells and incubated for 2 days at 37°C and then fixed with 4% paraformaldehyde for 30 min at RT, followed by staining with FITC conjugated polyclonal antibody against PEDV-N protein to visualize CPE in the cells. Serum samples were tested in triplicate, and neutralization titers were expressed as the reciprocal of the highest dilution of serum inhibiting CPE in all duplicate wells.

### 2.13. Piglet Infection Experiment

Ten 3-day-old colostrum-free piglets were obtained from sows that were not immunized with the PEDV vaccine. Sow and 3-day-old colostrum-free piglets were confirmed to be free of PEDV, PDCoV, PoRV, and TGEV through antibodies and viral nucleic acids test. Piglets were fed liquid milk and free drinking water, and the experiment was started after acclimation for 24 h. Piglets were randomly divided into two groups with 5 piglets in each group and housed in separate cages, which were PEDV CH/Yinchuan/2021 inoculation group and control group, respectively. The inoculated group was orally inoculated with P7 (passage 7) PEDV CH/Yinchuan/2021 at 10^4.5^ TCID_50_/ml (3 ml/piglet) as previously described [2, 38], while the control group was orally inoculated with 3 ml DMEM.

Clinical signs, including diarrhea severity, temperature, and weight were observed every 4 h during the day and every 12 h at night from the beginning to the end of the study. The clinical assessment including mental state and diarrhea severity were performed as previously described [34]. Fecal swabs were collected every 12 h until death. At the end of the study, all surviving piglets were humanely euthanized. At pathological necropsy, the duodenum, jejunum, ileum, cecum, and colon were fixed with 4% paraformaldehyde for histological analyses, and fresh tissues were stored at −80°C for viral detection.

### 2.14. Histopathology

Histopathology was performed as described previously [39]. Gastrointestinal tissues containing duodenum, jejunum, ileum, cecum, and colon were collected and fixed in paraformaldehyde. After fixation with 4% paraformaldehyde at RT for 48 h, the tissue specimens were processed and embedded in paraffin and subsequently stained with hematoxylin and eosin (H&E) (Beijing Solaris Science & Technology Co., Ltd., China).

### 2.15. Quantitative RT-PCR

Each fecal swab was diluted and homogenized with 1 ml sterile PBS at the indicated time points, centrifuged at 4,500 ×g for 10 min at 4°C, and 300 *μ*l supernatant was used to extract viral RNA. Equal quantities (0.1 g) of tissue samples were homogenized in 1 ml of sterile PBS, centrifuged at 4,500 ×g for 20 min at 4°C, and 300 *μ*l supernatant was used to extract RNA and synthesize cDNA as described above. The quantitative real-time PCR (qPCR) reactions were performed using the primers targeting M gene (PEDV-qPCR-F: 5′-TAT AAG GTT GCT ACT GGC GT-3′; PEDV-qPCR-R: 5′-CAT TGA CTG AAC GAC CAA CA-3′), and the qPCR reactions were carried out using SYBR qPCRMaster Mixture (DiNing, China) with the following reaction conditions: 94°C for 2 min, followed by 40 cycles of 94°C for 15 s and 55°C for 15 s. A standard plasmid based on the M gene was constructed in this study. Briefly, the PEDV CH/Yinchuan/2021 strain M gene was amplified using specific primers (PEDV-M-F: 5′-ACG GTT CTA TTC CCG TTG ATG-3′; PEDV-M-R: 5′-TAA ATG AAG CAC TTT CTC ACT ATC-3′) and RT-PCR product was cloned into the pMD19-T plasmid (Takara Bio, Inc., Otsu, Shiga, Japan) (pMD19-T-M). Standard curves were generated using 10-fold dilutions of pMD19-T-M (from 10^9^ to 10^1^) and a negative control (distilled water).

### 2.16. Statistical Analysis

Results are expressed as mean ± standard deviations (SD) and analyzed using the GraphPad Prism 9.4.1 software (GraphPad Software, Inc., La Jolla, CA, USA). Paired *t* test was performed to compare the two groups. One-way ANOVA followed by a *t*-test was conducted to compare multiple groups. Survival analysis was employed using the Kaplan–Meier method. Differences were defined as statistically significant at *p* <  0.05 (^*∗*^), *p*  <  0.01 (^*∗∗*^), *p*  <  0.001 (^*∗∗∗*^), and *p*  <  0.0001 (^*∗∗∗∗*^).

## 3. Results

### 3.1. Pathogen Identification

In July 2021, 9 intestinal contents and 47 fecal samples were derived from 7 adjacent sow farms in Yinchuan, Ningxia Hui Autonomous Region of China. To determine the causative agent of the diarrhea outbreaks, the diarrhoea-related enteric viruses including PEDV, PoRV, TGEV, and PDCoV were detected using RT-PCR. The 6 intestinal contents and 44 fecal samples were identified as PEDV-positive, whereas PoRV, TGEV, and PDCoV were not detected in all samples (data not shown). To obtain information about the genetic sequence information of these samples, we identified and sequenced the S gene in all PEDV-positive samples. Sequence alignment revealed that all sequences were almost identical (99.9-100%), suggesting the same PEDV strain was responsible for diarrhea outbreaks in affected sow farms.

### 3.2. Viral Isolation and Characterization

**Figure 1:**
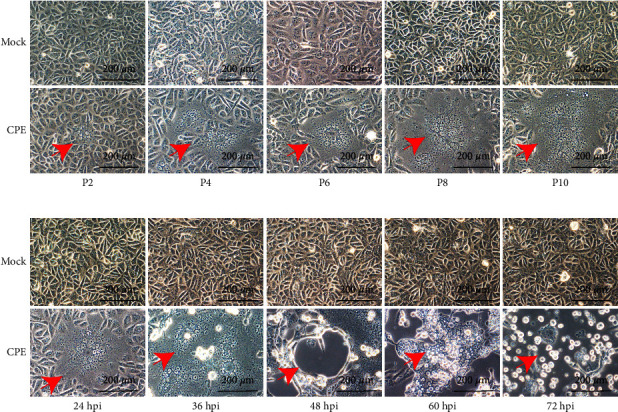
Cytopathic effects (CPE) of PEDV CH/Yinchuan/2021 in Vero cells. (a) Vero cells were infected or mock-infected with different passages PEDV CH/Yinchuan/2021; the PEDV-specific CPEs were observed at 24 hpi. The red arrow indicates CPE. (b) CPE appeared in different time points after PEDV CH/Yinchuan/2021 (P8) infection. The cells were detected employing a microscope at 200x magnification.

**Figure 2:**
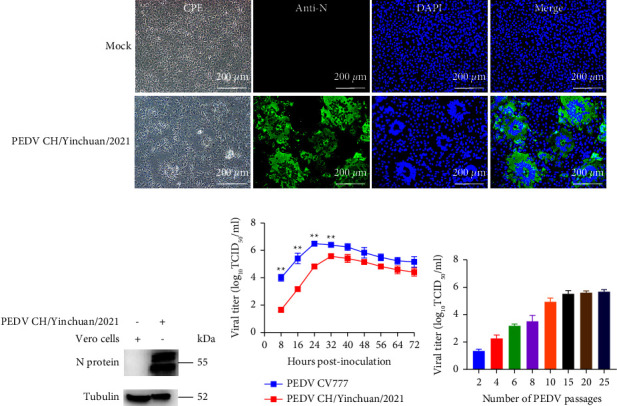
Identification of the PEDV CH/Yinchuan/2021. (a) Vero cells were infected or mock-infected with PEDV CH/Yinchuan/2021 (P10); the immunofluorescence assay (IFA) staining was performed with the polyclonal antibody against PEDV N protein at 24 dpi. The infected cells showed the formation of syncytia including different numbers of nuclei (green for antigens, blue for nuclei). The cells were observed using a fluorescence microscope at 200x magnification. (b) The western blot analysis was used to identify the N protein expression of PEDV CH/Yinchuan/2021 in Vero cells. (c) Vero cells were infected with PEDV CH/Yinchuan/2021 (P10) or CV777 and harvested at different time points, and the viral growth curve was determined. Data are shown as the mean ± SD of the results from three individual experiments. ^*∗∗*^*p*  <  0.01. (d) The PEDV CH/Yinchuan/2021 (different passages) titer was determined in infected Vero cells at 24 hpi.

The PEDV-PCR-positive samples including 6 intestinal homogenates and 44 fecal samples were attempted for virus isolation in Vero cells. A PEDV isolate named CH/Yinchuan/2021 was successfully isolated from the small intestine of a dead piglet in a sow farm. The obvious cytopathic effect (CPE) containing syncytium formation, multiple regional cell fusion, and eventual detachment appeared in infected Vero cells from passage 4 (P4) at 24 hpi (Figure 1(a)). To investigate whether CH/Yinchuan/2021 could proliferate in cell culture, the isolate was further passaged in Vero cells for 25 passages (P1–P25). Visible CPE was observed at P2 in 24 hpi, and CPE gradually became predominant in the later passages, including the progressively enlarging syncytium (Figure 1(a)). In addition, CPE had obvious changes in different time points at P8, including enlarging syncytium (36 h), cell detachment (48 h), cell vacuoles (60 h), round and shrink (72 h), and many floating cells were observed in the medium (72 h) (Figure 1(b)). To confirm whether the occurrence of virus propagation during passages, the infectivity of the CH/Yinchuan/2021 strain (P10) was assessed using IFA staining and western blot (WB) against the PEDV N protein polyclonal antibody. As shown in Figure 2(a), the specific staining of the PEDV-N protein was obviously distributed in the cytoplasm with syncytial cells, not in the nucleus. In contrast, no CPE and green signal were detected in the mock-infected cells. WB analysis also further confirmed PEDV-N-protein expression in the PEDV-infected Vero cells, but not in the mock-infected cells (Figure 2(b)). Moreover, the growth kinetics showed that the proliferative ability of the isolate at P10 was obviously weaker than that of the classical strain CV777 in Vero cells at 24 hpi (*p*  <  0.01), and the peak viral titer of CH/Yinchuan/2021 strain (10^5.58^ TCID_50_/ml at 32 hpi) was lower than that of the CV777 stain (10^6.5^ TCID_50_/ml at 24 hpi) (Figure 2(c)). During the first 10 serial passages, the viral titer ranged from 10^1.3^ to 10^4.83^ TCID_50_/ml at 24 hpi, whereas it was determined to be 10^5.67^ TCID_50_/ml in the P25 (Figure 2(d)).

### 3.3. Nucleotide Sequence Analysis of the CH/Yinchuan/2021

To elucidate the characteristics of the PEDV CH/Yinchuan/2021 strain, the entire genome sequence of CH/Yinchuan/2021 was obtained by next-generation sequence technology and deposited in the GenBank under accession number OP480878. A total of 28,038 nucleotides (nt) were determined for PEDV CH/Yinchuan/2021 strain, excluding the poly (A) tail. The organization that of other PEDV genomes previously sequenced with 5′UTR-ORF1a/1b-S-ORF3-E-M-N-3′UTR [9, 13]. We first carried out “Nucleotide BLAST” on the whole genome of CH/Yinchuan/2021 at NCBI and selected the top 50 strains for homology analysis. A comparison to 50 PEDV strains available in GenBank shared 98.8%–99.2% nt identity, with the highest nt identity (99.2%) with Chinese PEDV isolate SD2020 (GenBank accession No. OL762456). Subsequently, compared with 77 reference strains of different genotypes (Table S1), the full-gene of CH/Yinchuan/2021 isolate exhibited 96.4–97.2%, 96.8-96.9%, 98.3–98.7%, 97.7–99.2%, and 98.3–98.8% with reference GIa, GIb, S-INDEL, GIIa, and GIIb, respectively (Table 1). Note that, the S gene nt sequence showed the most diverse gene among the reference strains, and the GII strains shared a slightly higher percent identity than that of the GI and S-INDEL strains (Table 1). In addition, the S1 subunit in all genotypes exhibited lower nt percent identities compared with the S2 subunit (Table 1).

### 3.4. Phylogenetic and Recombination Analysis

**Figure 3:**
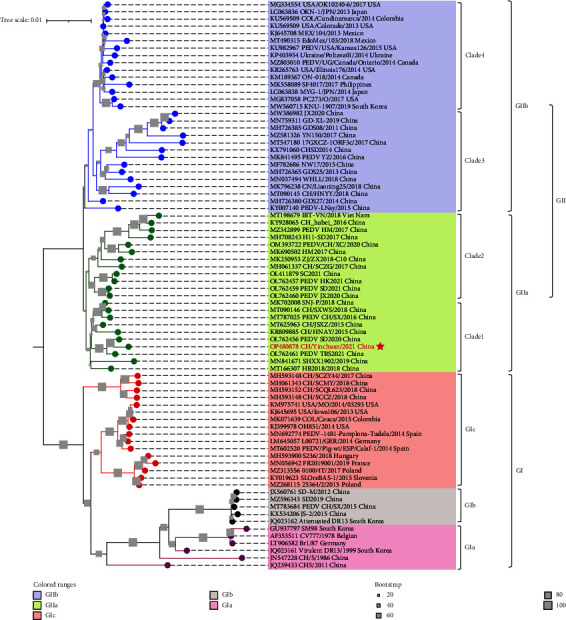
Phylogenetic analysis of the PEDV based on the S gene. The nucleotide sequences of reference PEDV strains worldwide are downloaded from GenBank. The tree was constructed using the MEGA 7 software program with the neighbour joining method and bootstrap methods estimated for 1,000 replications. The GenBank accession numbers, strain names, collection date, and countries are shown in the trees. Different subgroups were marked by different colours. GIa, GIb, GIc, GIIa, and GIIb were marked pink, grey, red, green, and blue, respectively. The grey square icon at the branch represents bootstrap values greater than 20% of 1000 replicates. The PEDV CH/Yinchuan/2021 was marked in red.

To clarify the evolution relationship between CH/Yinchuan/2021 and other PEDV strains globally, phylogenetic analyses were performed based on the nt sequences of the S gene of PEDV isolate identified in this study and 77 references PEDV strains (Table S1). As shown in Figure 3, the phylogenetic tree revealed all the PEDV strains distinctly clustered into two major groups, GI and GII, which were clearly separated into the GIa, GIb, and GIc, or GIIa and GIIb subgroups, respectively. All GIa strains (*n* = 6) included attenuated vaccine strain CV777 and virulent DR13, and the CH/S strain, originally isolated in China, formed an independent branch within this subgroup (Figure 3). The GIb subgroup (*n* = 5) contained attenuated DR13 strain and several Chinese field isolates since 2012. The GIc (*n* = 16) was assigned as the S-INDEL subgroup, which showed two branches within three clades, one including American, Colombian, German, and Spanish strains during 2013–2015, and the other containing only European national strains such as Poland, France, Slovenia, and Hungary since 2015. However, the S-INDEL strains from China formed a single branch within one clade, which was separate for S-INDEL strains from other countries (Figure 3). Notably, the GIIa subgroup (*n* = 22) was the circulating strains identified in China since 2015 except for a Vietnamese isolate (IBT-VN, GenBank No. KY928065), and obviously divided into two clades (Clade 1 and Clade 2). The CH/Yinchuan/2021 isolate belonged to the GIIa subgroup, forming an independent branch with 2020-2021 isolates from China within Clade 1. The GIIb subgroup (*n* = 29) also was classified into two clades (Clade 3 and Clade 4). However, Clade 3 only contained pandemic strains isolated in China from 2011 to 2020, which were obviously different from the sources of strains circulating in North America, South America, and Europe countries within Clade 4, especially the isolates from the USA (5/15).

**Figure 4:**
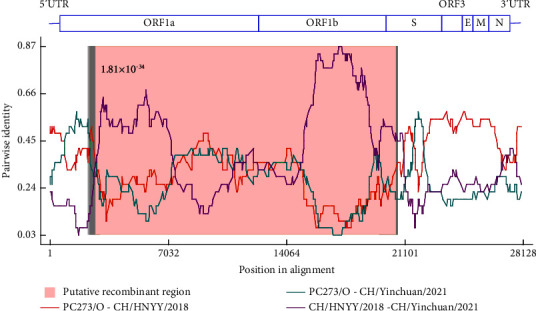
Genome recombination analysis of CH/Yinchuan/2021. Recombination analysis was determined using RDP4 software. The putative recombinant region was highlighted with a pink background and *p*-value is shown next to it. Comparisons between the parent isolates (PC273/O-CH/HNYY/2018) were shown in the red line, comparisons between CH/Yinchuan/2021 and its major parent (PC273/O) or minor parent (CH/HNYY/2018) as shown in cyan line and purple line, respectively. The upper schematic diagram represented the genome organization of PEDV CH/Yinchuan/2021.

To further analyze potential recombination in CH/Yinchuan/2021 isolate during evolution, we performed recombination examination and analysis employing RDP4 software by the whole genome sequence of PEDVs (*n* = 77) (Table S1). Strikingly, CH/Yinchuan/2021 was analyzed as a recombinant originated from the PC273/O strain (USA) (major parent) and CH/HNYY/2018 strain (China) (minor parent), with a big recombinant region spanning ORF1a (nt 2716 without gaps) and S1 the 5′ proximal part of S1 (nt 19570–20711 without gaps) (Figure 4), supported by more than five recombination detection algorithms (Table 2).

### 3.5. Analysis of PEDV S Protein

**Figure 5:**
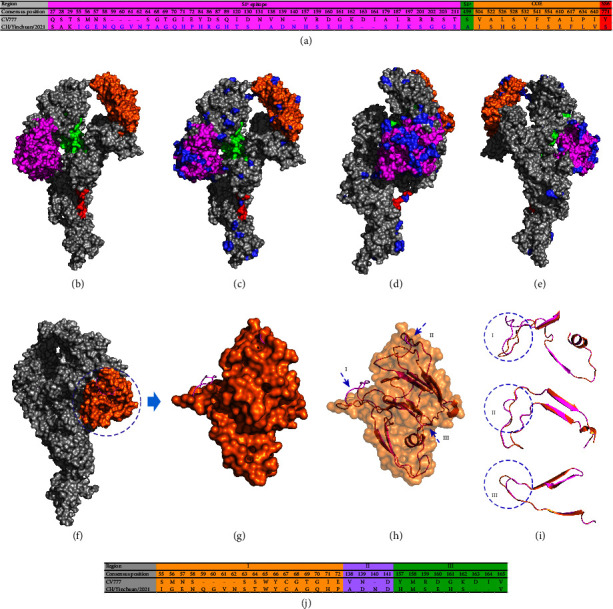
S protein analysis of CH/Yinchuan/2021 strain. (a) The CH/Yinchuan/2021 strain amino acid (aa) differences relative to the consensus of the PEDV CV777 strain. The S1^0^, S1^A^, COE, and SS6 epitopes are highlighted magenta, green, orange, and red, respectively. (b) S protein model of CV777 strain. (c) S protein model of CH/Yinchuan/2021 strain. (d and e) The S protein structure of CH/Yinchuan/2021 is rotated 90° and 180° relative to (c), respectively. The S1^0^, S1^A^, COE, and SS6 epitope domains are highlighted magenta, green, orange, and red, respectively. Grey represents the epitope region without aa difference and the nonepitope region. The mutant aa residues of the CH/Yinchuan/2021 S protein are shown as surface by blue. (f) Comparison of the PEDV S protein structure presented CH/Yinchuan/2021 (shown as a cartoon by magenta) with CV777 strain (shown as surface by gray). The dashed circle region is the S1^0^ epitope model of CV777 strain shown as surface by orange. (g and h) Comparison of the S1^0^ epitope structure between CH/Yinchuan/2021 (shown as a cartoon by magenta) and CV777 strain (shown as surface by orange). The blue arrows indicate the location of the structural change (I, II, and III). (i) Structural changes of S1^0^ epitope between CH/Yinchuan/2021 (magenta) and CV777 (orange) strain. (j) Structural changes of S1^0^ epitope in aa residue regions.

Compared with the CV777 vaccine strain, the CH/Yinchuan/2021 exhibited several insertions, deletions, and substitutions, including 5-aa insertion (^59^QGVN^62^ and ^140^N), 2-aa deletion (^163^DI^164^), and 84-aa substitutions. To further investigate the aa differences in the S protein between the CH/Yinchuan/2021 and CV777 strain, six neutralizing epitopes of the CH/Yinchuan/2021 strain (S1^0^, S1^A^, COE, SS2, SS6, and 2C10) were analyzed in this study. As shown in Figure 5(a), the CH/Yinchuan/2021 strain identified 41, 1, 11 and 1 aa differences within S1^0^, S1^A^, COE, and SS6 epitope domains, respectively, and no differences were found in the SS2 and 2C10 epitopes. In addition, 38 aa substitutions were found between CH/Yinchuan/2021 and CV777 strains in the nonepitope regions (Table S3). Importantly, multiple alignments of S protein aa sequences (*n* = 78) uncovered that the CH/Yinchuan/2021 strain has 6 unique aa substitutions, including 1 (T29K), 2 (V512I and P646L) and 3 (V247I, V318G, and V354I) in S1^0^, COE epitope and nonepitope region, respectively (Table S3). Furthermore, we found a highly variable region at the S1 NTD (1–378 aa) between the GI and GII strains, especially in the S1^0^ epitope region (Table S3). Remarkably, these residue substitutions in S protein, whether epitopes or nonepitopes, occurred on the surface of its structure, especially the insertion and deletion of multiple consecutive residues at the S1^0^ epitope (Figures 5(b)–5(e)). Further structural analysis of S protein uncovered that the CH/Yinchuan/2021 exhibited three obvious structural changes in residues 55–72, 138–141, and 157–165 located in the S1^0^ epitope region (Figures 5(f)–5(j)).

### 3.6. Cross-Neutralization Analysis of CV777 and CH/Yinchuan/2021 Strains

**Figure 6:**
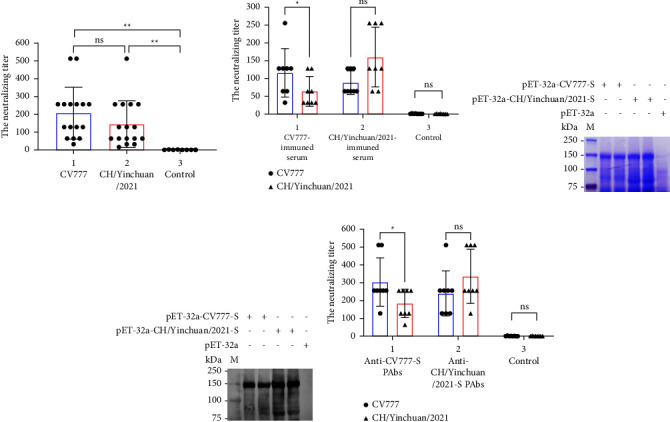
Cross-neutralization analysis of CV777 and CH/Yinchuan/2021 strains. (a) Serum samples (*n* = 16) were collected from sows inoculated with the CV777 strain, and the neutralizing antibodies against CV777 and CH/Yinchuan/2021 strains were tested using VNT. In the control group, serum samples (*n* = 8) were collected from PEDV-negative sows. (b) Mice were immunized with inactivated PEDV CV777, CH/Yinchuan/2021, and PBS (control), the neutralizing antibody titers against CV777 and CH/Yinchuan/2021 strains were detected in collected serum (*n* = 8). (c) The S protein expression of pET-32a-CV777-S and pET-32a-CH/Yinchuan/2021-S were analyzed by SDS-PAGE with Coomassie brilliant blue R250. The pET-32a vector was used as a control for analysis. (d) The purified S proteins were detected by Western blotting with anti-His mouse Mab. (e) Mouse polyclonal antibodies (PAbs) were prepared by immunizing mice with purified pET-32a-CV777-S or pET-32a-CH/Yinchuan/2021-S recombinant protein (*n* = 8), the levels of neutralizing antibody against PEDV CV777 and CH/Yinchuan/2021 strains were detected. ^*∗∗*^*p*  <  0.01; ^*∗*^*p*  <  0.05; ns, not significant.

To evaluate the levels of neutralizing antibodies against PEDV in sow farms inoculated with CV777 strain, serum samples were collected from sows and tested for neutralizing antibodies using VNT. As shown in Figure 6(a), the anti-CV777 neutralizing antibody titers range from 32 to 512 (average titer 206), and the titer of neutralizing CH/Yinchuan/2021 is 145, nearly 1.4-fold lower than that of the titer of anti-CV777, while there were no differences in neutralizing antibody between CV777 and CH/Yinchuan/2021 (*p*  >  0.05). To further verify the cross-neutralizing activity, mice were immunized with inactivated CV777 and CH/Yinchuan/2021, and then the cross-neutralizing antibody titers were analyzed. Our data showed that the titer of anti-CV777 in CV777-immuned serum was significantly higher than that of anti-CH/Yinchuan/2021 (*p*  <  0.05) (Figure 6(b)). Although there was no significant difference in the ability of CH/Yinchuan/2021-immuned serum to neutralize CV777 and CH/Yinchuan/2021 strains (*p*  >  0.05), the titer of anti-CH/Yinchuan/2021 was nearly 2-fold higher than that of anti-CV777 (Figure 6(b)). These data indicated that the antigenicity of CH/Yinchuan/2021 had some changes relative to the classical strain CV777. To verify whether the antigenicity change is caused by the difference between the S protein of CH/Yinchuan/2021 and CV777 strain, the cross-neutralization antibody titer against the S protein mouse polyclonal antibody (PAbs) was evaluated. As shown in Figure 6(c), the S protein with an expected molecular weight of 150 kDa was found by SDS-PAGE, and the expressed S proteins of CH/Yinchuan/2021 and CV777 strains were further confirmed by western blotting (Figure 6(d)). Neutralizing antibody levels revealed that the ability of anti-CV777-S PAbs to neutralize CV777 was significantly higher than that of CH/Yinchuan/2021 (*p*  <  0.05) (Figure 6(e)). Notably, anti-CH/Yinchuan/2021-S PAbs showed no difference in neutralizing CV777 and CH/Yinchuan/2021 strains (*p*  >  0.05), whereas the average titer neutralizing CH/Yinchuan/2021 strain (336) was 1.4-fold higher than that of CV777 strain (240) (Figure 6(e)).

### 3.7. Pathogenicity of PEDV CH/Yinchuan/2021

**Figure 7:**
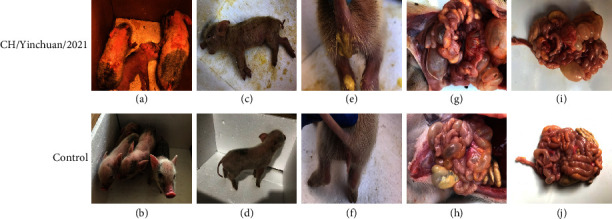
Clinical assessment and necropsy examination of piglets challenged with PEDV CH/Yinchuan/2021. The 3-day-old piglets were infected or mock-infected orally with the CH/Yinchuan/2021, and clinical symptoms (a, c, and e) and necropsy examination (g and i) were observed in the infected group and mock group (b, d, f, h, and j).

**Figure 8:**
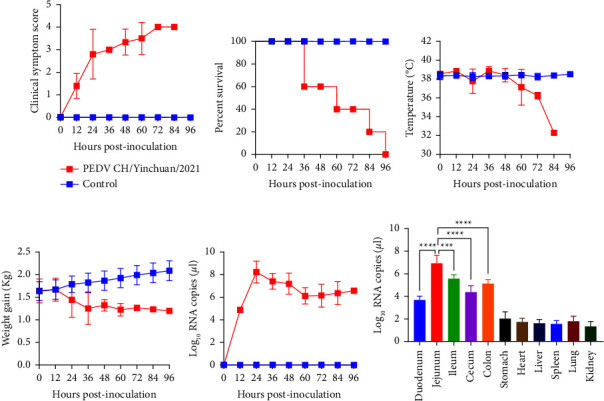
Pathogenicity, viral shedding, and distribution analysis. The clinical symptom scores (a), percent survival (b), rectal temperature (c), and body weight (d) were determined in each group after PEDV CH/Yinchuan/2021 infection. (e) Virus shedding was detected in rectal swabs at different time points. (f) Virus distribution was determined in different tissues after a necropsy examination. ^*∗∗∗*^*p*  <  0.001 and ^*∗∗∗∗*^*p*  <  0.0001.

To investigate the pathogenicity of PEDV CH/Yinchuan/2021, 3-day-old piglets were infected orally with the CH/Yinchuan/2021. At 18 hpi, 4/5 piglets began to exhibit semisolid feces and vomiting in the challenge group, and all piglets at 24 hpi presented with serious PED-symptoms characterized by watery diarrhea, lethargy, loss of appetite, huddle, and shortness of breath, but vomiting was rare (Figures 7(a)–7(e) and 8(a)). In the challenge group, yellow and foul-smelling watery feces remained in the perianal region of piglets (Figure 7(e)). No clinical symptoms were observed in the control group (Figures 7(b), 7(d), and 7(f)). Two infected piglets died with severe dehydration at 36 hpi, the remaining three piglets died successively at 60 and 96 hpi (Figure 8(b)). In addition, the rectal temperature of challenged piglets did not change significantly but significantly decreased 12 h before death, and the body weight also gradually decreased compared with the control group (Figures 8(c) and 8(d)).

**Figure 9:**
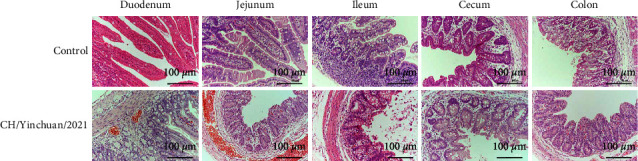
Histopathological analysis. The piglets were infected or mock-infected orally with the CH/Yinchuan/2021, and the pathological changes of different tissues including duodenum, jejunum, ileum, cecum, and colon were identified by hematoxylin and eosin (H&E) staining (100x magnification).

Viral RNA was detected in all rectal swab samples (5/5) form challenged piglets at 12 hpi, and reached the peak of 8.22 log_10_ RNA copies/*μ*l at 24 hpi, followed by 6.12–8.22 log_10_ RNA copies/*μ*l at 24–96 hpi (Figure 8(e)). In addition, our data showed that the main colonization site of PEDV was the intestine, containing duodenum, jejunum, ileum, cecum, and colon, and peak viral RNA titers reached up to 6.92 log_10_ RNA copies/*μ*l in the jejunum, this titer was obviously higher than that in other intestinal tissues (*p*  <  0.001) (Figure 8(f)). The low-level RNA expression was also detected in the stomach, heart, liver, spleen, lung, and kidney (Figure 8(f)). No viral RNA was detected in all samples of uninfected piglets (Figure 8(e)). Necropsy examinations showed that the small intestinal tract (duodenum to colon) was distended, transparent, relaxed, lacked elasticity, and filled with yellow fluid (Figures 7(g) and 7(i)), whereas no such changes were observed in the control group (Figures 7(h) and 7(j)). The stomach is filled with curdled milk, mesenteric congestion and enlarged mesenteric lymph nodes (Figures 7(g) and 7(i)). Histopathologic analysis revealed severe villous atrophy in the duodenum, jejunum, ileum, cecum, and colon (Figure 9), the duodenum and jejunum presented with bleeding, and the fusion and sloughing were observed in the ileum and cecum (Figure 9). No lesions were found in uninfected piglets (Figure 9).

## 4. Discussion

Originally identified in the United Kingdom, PEDV is now a global pathogen, especially since 2010 when highly pathogenic variants have emerged in many countries worldwide, posing a huge economic threat to the global pig industry [9, 17, 40, 41]. Before 2010, inactivated and attenuated vaccines based on CV777 played a major role in the prevention and control of PEDV in China. Since then, however, multiple studies have shown that the CV777 vaccine failed to provide complete protection against highly pathogenic variants of PEDV, as PEDV has unexpectedly devastated many swine farms vaccinated with the vaccine [2, 7, 8]. In July 2021, a serious diarrhea occurred in several adjacent sow farms vaccinated with the CV777 vaccine strain in Yinchuan, China, which was characterized by severe vomiting, lethargy, and moderate diarrhea in sows, watery fetid diarrhea, vomiting, dehydration in suckling piglets, and 100% mortality. PEDV was finally confirmed as the only main causative agent by excluding other possible pathogens including PoRV, TGEV, and PDCoV. In the present study, 9 intestinal contents and 47 fecal samples were collected from 7 affected sow farms, we successfully isolated a PEDV CH/Yinchuan/2021 from the small intestinal contents of dead piglets using Vero cells as previously described [33]. Although clinical samples were collected from several sow farms, we unfortunately isolated only one isolate (success rate, 2%), as the isolation of PEDV is affected by many factors, including sample type, trypsin concentration, and cell susceptibility to PEDV [42, 43]. In this study, the PEDV was not isolated from all fecal samples, but from a single intestinal sample, suggesting that it was easier to isolate the virus from intestinal samples than from fecal samples, which is consistent with previous reports [42, 44]. Therefore, the isolation methods need to be improved so that more isolates can be obtained in the lab. Previous studies reported that other isolates showed visible CPE until P7 [33, 43], while the isolate CH/Yinchuan/2021 began to show CPE from the P2, and became more evident with subsequent passages of Vero cells. This discrepancy may be attributable to the isolation methods or cell susceptibility to PEDV to different isolates. Our data uncovered that the infectivity of the CH/Yinchuan/2021 isolate at P10 was significantly weaker than that of the CV777 in Vero cells at 24 hpi. However, this was in contrast to the results of Zhang et al., who reported the proliferation capacity of the isolate CH/JX/01 was higher than that of CV777 [45]. This difference may be related to the characteristics of the isolate itself and the number of virus passages. To investigate the characteristics of CH/Yinchuan/2021, the full genome sequence was obtained by next-generation sequence technology. We found that CH/Yinchuan/2021 isolate exhibits 96.4–97.2%, 96.8–96.9%, 98.3–98.7%, 97.7–99.2%, and 98.3–98.8% with reference GIa, GIb, S-INDEL, GIIa, and GIIb, respectively, indicating the CH/Yinchuan/2021 has more similarities with GII than GI and S-INDEL strains.

To further analyze the evolutionary relationship between PEDV CH/Yinchuan/2021 and other isolates globally, a phylogenetic tree was constructed based on S-gene sequences in this study. All the analyzed PEDV strains were distinctly clustered into two major groups, GI and GII, and further were allocated into five subgroups (GIa, GIb, GIc, GIIa, and GIIb). GIa and GIb are consistent with previous phylogenetic topology [17, 40], including attenuated vaccine strains and field strains originally isolated in some countries. However, the GIc (S-INDEL) has distinct temporal and regional branches, all the Chinese S-INDEL strains showed a single branch within one clade, other analyzed S-INDEL strains formed two clades, one containing strains from European countries such as Poland, France, Slovenia, and Hungary since 2015, and the other containing strains from the United States, Colombia, Germany, and Spain during 2013–2015. These data indicate that the S-INDEL strains may have spread and continued to evolve in European and North American countries. Remarkably, GIIa and GIIb obviously contain two clades each (Clade 1, Clade 2, Clade 3, and Clade 4), Clade 1 and Clade 2 form two groups circulating GIIa strains in China since 2015 except a Vietnamese isolate (IBT-VN, GenBank No. KY928065). The CH/Yinchuan/2021 isolate belonged to the GIIa subgroup and formed an independent branch with Chinese isolates from 2020–2021 within Clade 1. Notably, Clade 3 included only pandemic strains isolated in China since 2011, which was significantly different from strains circulating in North America, South America, and European countries in Clade 4, especially strains from the USA (5/15). These phylogenetic data indicate that there are regional differences among global variant PEDV strains, especially the continuous evolution of circulating strains in China, forming multiple lineages that are different from those in other countries. Undoubtedly, the continuous evolution of PEDV in different regions over time, which will bring more challenges to global prevention and control.

Recombination is considered to be a crucial means in viral evolution, especially for coronaviruses, leading to changes in pathogenicity, host range, and transmission routes [46]. Recombination among viruses poses a huge potential threat to human and animal life as many human coronaviruses originate from animal reservoirs [47, 48]. The PEDVs currently circulating in China including GI, GII, and S-INDEL strains, which are also constantly recombining, and then new strains are constantly emerging. In the present study, the CH/Yinchuan/2021 strain was detected as a recombinant with a big recombinant region spanning ORF1a and S1. Interestingly, CH/Yinchuan/2021 was derived from a major parent (PC273/O strain) in the United States and a minor parent (CH/HNYY/2018 strain) in China. A recent study revealed that the United States became an important source for the introduction of PEDV to many countries, including Japan, Korea, China, and Mexico after an initial introduction out of China, and the live swine trade played an important role in the dispersal of PEDV on a global scale [49]. These data suggest that the isolate CH/Yinchuan/2021 may have been recombined from strains circulating in China and the United States through the global trade in live swine and swine-related products. The discovery of this virus indicates that PEDV strains circulating in farms in China are becoming increasingly complex, and it is necessary to conduct a molecular epidemiological investigation and evolutionary analysis of PEDV strains circulating locally in China.

The PEDV S protein is the most diverse protein, and aa changes can result in virus variation and influence virulence, especially in the S1 region, its mutations alter viral tropism and pathogenicity in piglets [27, 50, 51]. In the present study, the CH/Yinchuan/2021 isolate shared 5-aa insertion (^59^QGVN^62^ and ^140^N) and 2-aa deletion (^163^DI^164^) compared with the CV777 strain, which is consistent with previous results [9, 17], suggesting these typical variants are considered markers for distinguishing non-S-INDEL from S-INDEL strains [14, 16]. In addition, multiple neutralizing epitopes in PEDV S protein have been identified containing S1^0^, S1^A^, COE, SS2, SS6, and 2C10, which can induce robust neutralizing antibodies against PEDV [23–26, 37]. Our results revealed that the CH/Yinchuan/2021 harbored 41, 1, 11, and 1 aa differences in S1^0^, S1^A^, COE, and SS6 epitope domains, respectively, and 38 aa substitutions in the nonepitope region compared with CV777 strain. More importantly, these aa substitutions in S protein occur on the surface of its structure. Therefore, these mutations are considered to be markers for emerging global PEDV strains, which may cause the CV777 vaccine to be unable to combat current circulating strains [29, 50]. Furthermore, multiple alignments of S protein aa sequences (*n* = 78) showed that the CH/Yinchuan/2021 presented 6 unique aa substitutions, such as 1 (T29K), 2 (V512I and P646L) and 3 (V247I, V318G, and V354I) in S1^0^, COE epitope, and nonepitope region, respectively. The specific mechanism of its substitutions needs further study. S1^0^ epitope was identified as a sialoglycoconjugates-binding domain, which localizes to the S1 NTD (20–220 aa) [22]. A study uncovered that a monoclonal antibody derived from the S1^0^ epitope of non-S-INDEL strain could not or partially cross-neutralize against CV777 and S-INDEL strains, independent aa substitution in S1^0^ region can also result in antigenic variation [37]. Consistent with the previous studies [17, 44], we also found a highly variable region (1–378 aa) in the S1 NTD, especially the S1^0^ epitope region between the GI and GII strains. In addition, S protein model analysis indicated that the CH/Yinchuan/2021 isolate showed three structural changes in the S1^0^ epitope region located at residues 55–72, 138–141, and 157–165. The effect of these antigenic drifts on host immunogenicity and pathogenicity needs to be further studied.

Although studies have confirmed that the classic vaccine strain CV777 is less effective in protecting against the highly pathogenic PEDV variants, the inactivated and attenuated PEDV vaccines based on CV777 are still used in some pig farms in China [2, 7, 8]. In the present study, we assessed the level of CV777 vaccine immunity in sow farms which experienced a PEDV invasion in Yinchuan, China, in 2021. Our data showed that high levels of neutralizing antibodies against CV777 were detected in immunized sows. In addition, the titer of ani-CV777 is nearly 1.4-fold higher than that of ani-CH/Yinchuan/2021, whereas the ability of some samples to neutralize CH/Yinchuan/2021 is higher than that of CV777, which may be related to the infection of CH/Yinchuan/2021 in pig farms. Moreover, experiments on mice further verified that the ability of CV777-immuned serum to neutralize CH/Yinchuan/2021 was significantly decreased compared with the CV777 strain. Meanwhile, we also found that the cross-neutralizing reaction of CH/Yinchuan/2021-immuned serum was higher than that of CV777. These data suggest that PEDV CH/Yinchuan/2021 had some antigenic differences with the CV777 strain. To further verify whether the mutation of the S protein changes the antigenicity of the PEDV CH/Yinchuan/2021, two mouse PABs of PEDV S protein were prepared and their cross-neutralization titers were determined. Our results showed that the titer of anti-CV777-S PAbs against CH/Yinchuan/2021 was significantly lower than that of CV777, while the titer of anti-CH/Yinchuan/2021-S PAbs against CV777 also decreased slightly than that of CH/Yinchuan/2021, suggesting that differences in the S protein between CH/Yinchuan/2021 and CV777 alter the immunogenicity, thereby resulting in a different neutralization profile. These data further confirmed that the commercially available CV777 does not effectively neutralize the highly pathogenic PEDV variant, which may be due to the mutation of the neutralizing epitope of the S protein leading to the decreased protective efficacy of the CV777 vaccine. It also reminds us that the CV777 vaccine should be used cautiously in China, and it is urgent to develop a new vaccine based on the variants as the parent strain.

Since 2010, the emerging PEDV strains globally, whether GIIa or GIIb epidemic strains have been highly pathogenic to newborn piglets [4, 11, 34, 44]. In addition to typical clinical symptoms, PEDV is also characterized by rapid onset, rapid transmission, and rapid death, leading to almost all the deaths of piglets in one or several pig farms, and various treatment measures are insignificant. In this study, we observed that piglets infected with CH/Yinchuan/2021 strain began to exhibit symptoms at 18 hpi, began to die at 36 hpi, and all died at 96 hpi. These data uncovered that the PEDV CH/Yinchuan/2021 is highly pathogenic to suckling piglets. However, there were slight differences from the previously reported GII variants, the HM2017 strain caused mild diarrhea in piglets starting at 12 hpi, with 100% mortality at 84 hpi [34], PEDV SH developed mild diarrhea at 18 hpi, began to die at 69 hpi, with 100% mortality at 84 hpi [2]. Moreover, we found that virus shedding began at 12 hpi and peaked at 24 hpi, and the virus mainly colonized jejunum. These phenomena could provide some insights: why does PEDV proliferate so rapidly in the intestinal tract? What factors in the intestinal environment provide opportunities for PEDV? Answering these questions may be beneficial to the prevention or treatment of PEDV. A recent study revealed that a variant PEDV is not limited to the intestinal tract, but also disrupts the respiratory system, especially the lungs, indicating an evolutionary change in PEDV tropism as an enteric pathogen, which deserves our great attention [41].

In conclusion, a PEDV CH/Yinchuan/2021 strain was isolated from a large-scale PEDV-infected sow farm vaccinated with the CV777 vaccine in Yinchuan, China. Phylogenetic analysis revealed CH/Yinchuan/2021 isolate belonged to the major circulating GIIa in China. Multiple alignments of S protein aa sequences demonstrated that CH/Yinchuan/2021 showed multiple aa insertions, deletions, and substitutions in epitope and contained several unique aa substitutions. Cross-neutralization analysis confirmed that differences in the S protein between CH/Yinchuan/2021 and CV777 altered the antigenicity, leading to different neutralization profiles. In addition, we found that CH/Yinchuan/2021 is a highly pathogenic PEDV strain with 100% mortality in 3-day-old piglets. These data are helpful to understand the molecular characteristics, epidemiology, evolution, and antigenicity of circulating PEDV strains in China.

## Figures and Tables

**Table 1:** The nucleotide (nt) of CH/Yinchuan/2021 strain percent identity within GIa, GIb, S-INDEL, GIIa, and GIIb strains.

Genome/gene	All strains (*n* = 77)	GIa strains (*n* = 6)	GIb strains (*n* = 5)	S-INDEL strains (*n* = 15)	GIIa strains (*n* = 22)	GIIb strains (*n* = 29)
Complete genome	96.4–99.2%	96.4–97.2%	96.8–96.9%	98.3–98.7%	97.7–99.2%	98.3–98.8%
Spike (S)	93.4–99.5%	93.4–85.2%	93.5–93.6%	95.8%–96.4%	97.4–99.5%	96.7–98.5%
S1	91.3–99.4%	91.3–92.9%	91.4–98.4%	93.4–97.9%	97.2%–99.4%	96.8–98.0%
S2	95.5–99.8%	95.5–98.4%	96.2–96.3%	98.7–99.3%	97.8–99.8%	96.7–99.2%

**Table 2:** Recombination detection algorithms of CH/Yinchuan/2021 strain by RDP.

Methods	Seqs detected in	Average *p*-value
RDP	7	1.810 × 10^−34^
GENECOVN	5	5.670 × 10^−22^
BootScan	2	6.642 × 10^−15^
MaxChi	6	5.164 × 10^−10^
Chimaera	7	9.117 × 10^−07^
SiScan	6	8.277 × 10^−05^
3Seq	11	2.414 × 10^−12^

## Data Availability

The whole genome sequence of the PEDV CH/Yinchuan/2021 strain is openly available in the NCBI GenBank Accession number OP480878.

## Supplementary Materials

Supplementary Materials Table S1 The reference PEDV strains used in this study. Table S2 Sequences and primer pair characteristics in this study. Table S3 Multiple alignments of S protein aa sequences (*n* = 78) in this study.

## References

[1] Jung K., Saif L. J., Wang Q. (2020). Porcine epidemic diarrhea virus (PEDV): an update on etiology, transmission, pathogenesis, and prevention and control. *Virus Research*.

[2] Wang X. W., Wang M., Zhan J. (2020). Pathogenicity and immunogenicity of a new strain of porcine epidemic diarrhea virus containing a novel deletion in the N gene. *Veterinary Microbiology*.

[3] Woo P. C. Y., Huang Y., Lau S. K. P., Yuen K. Y. (2010). Coronavirus genomics and bioinformatics analysis. *Viruses*.

[4] Lin C. M., Saif L. J., Marthaler D., Wang Q. (2016). Evolution, antigenicity and pathogenicity of global porcine epidemic diarrhea virus strains. *Virus Research*.

[5] Pensaert M. B., de Bouck P. (1978). A new coronavirus-like particle associated with diarrhea in swine. *Archives of Virology*.

[6] Chen J., Wang C., Shi H. (2010). Molecular epidemiology of porcine epidemic diarrhea virus in China. *Archives of Virology*.

[7] Li W., Li H., Liu Y. (2012). New variants of porcine epidemic diarrhea virus, China, 2011. *Emerging Infectious Diseases*.

[8] Wang D., Fang L., Xiao S. (2016). Porcine epidemic diarrhea in China. *Virus Research*.

[9] Chen J., Liu X., Shi D., Shi H., Zhang X., Feng L. (2012). Complete genome sequence of a porcine epidemic diarrhea virus variant. *Journal of Virology*.

[10] Wang J., Zhao P., Guo L. (2013). Porcine epidemic diarrhea virus variants with high pathogenicity, China. *Emerging Infectious Diseases*.

[11] Stevenson G. W., Hoang H., Schwartz K. J. (2013). Emergence of porcine epidemic diarrhea virus in the United States: clinical signs, lesions, and viral genomic sequences. *Journal of Veterinary Diagnostic Investigation*.

[12] Wang L., Byrum B., Zhang Y. (2014). New variant of porcine epidemic diarrhea virus, United States, 2014. *Emerging Infectious Diseases*.

[13] Chen Q., Li G., Stasko J. (2014). Isolation and characterization of porcine epidemic diarrhea viruses associated with the 2013 disease outbreak among swine in the United States. *Journal of Clinical Microbiology*.

[14] Hanke D., Pohlmann A., Sauter-Louis C. (2017). Porcine epidemic diarrhea in Europe: in-detail analyses of disease dynamics and molecular epidemiology. *Viruses*.

[15] Huang Y. W., Dickerman A. W., Piñeyro P. (2013). Origin, evolution, and genotyping of emergent porcine epidemic diarrhea virus strains in the United States. *mBio*.

[16] Su M., Li C., Qi S. (2020). A molecular epidemiological investigation of PEDV in China: characterization of co-infection and genetic diversity of S1-based genes. *Transboundary and Emerging Diseases*.

[17] Tian Y., Yang X., Li H. (2021). Molecular characterization of porcine epidemic diarrhea virus associated with outbreaks in southwest China during 2014-2018. *Transboundary and Emerging Diseases*.

[18] Brian D. A., Baric R. S. (2005). Coronavirus genome structure and replication. *Current Topics in Microbiology and Immunology*.

[19] Li F. (2016). Structure, function, and evolution of coronavirus spike proteins. *Annual review of virology*.

[20] Walls A. C., Tortorici M. A., Bosch B. J. (2016). Cryo-electron microscopy structure of a coronavirus spike glycoprotein trimer. *Nature*.

[21] Hulswit R. J. G., de Haan C. A. M., Bosch B. J. (2016). Coronavirus spike protein and tropism changes. *Advances in Virus Research*.

[22] Li B., Du L., Yu Z. (2017a). Poly (D,L-lactide-co-glycolide) nanoparticle-entrapped vaccine induces a protective immune response against porcine epidemic diarrhea virus infection in piglets. *Vaccine*.

[23] Chang C. Y., Cheng I. C., Chang Y. C. (2019). Identification of neutralizing monoclonal antibodies targeting novel conformational epitopes of the porcine epidemic diarrhoea virus spike protein. *Scientific Reports*.

[24] Chang S. H., Bae J. L., Kang T. J. (2002). Identification of the epitope region capable of inducing neutralizing antibodies against the porcine epidemic diarrhea virus. *Molecules and Cells*.

[25] Okda F. A., Lawson S., Singrey A. (2017). The S2 glycoprotein subunit of porcine epidemic diarrhea virus contains immunodominant neutralizing epitopes. *Virology*.

[26] Cruz D. J. M., Kim C. J., Shin H. J. (2008). The GPRLQPY motif located at the carboxy-terminal of the spike protein induces antibodies that neutralize Porcine epidemic diarrhea virus. *Virus Research*.

[27] Li D., Li Y., Liu Y. (2021). Isolation and identification of a recombinant porcine epidemic diarrhea virus with a novel insertion in S1 domain. *Frontiers in Microbiology*.

[28] Sato T., Takeyama N., Katsumata A., Tuchiya K., Kodama T., Kusanagi K. I. (2011). Mutations in the spike gene of porcine epidemic diarrhea virus associated with growth adaptation in vitro and attenuation of virulence in vivo. *Virus Genes*.

[29] Vlasova A. N., Marthaler D., Wang Q. (2014). Distinct characteristics and complex evolution of PEDV strains. *Emerging Infectious Diseases*.

[30] Qi M., Zambrano-Moreno C., Pineda P. (2021). Several lineages of porcine epidemic diarrhea virus in Colombia during the 2014 and 2016 epidemic. *Transboundary and Emerging Diseases*.

[31] Liu B. J., Zuo Y. Z., Gu W. Y. (2018). Isolation and phylogenetic analysis of porcine deltacoronavirus from pigs with diarrhoea in Hebei province, China. *Transboundary and emerging diseases*.

[32] Tan L., Li Y., He J. (2020). Epidemic and genetic characterization of porcine epidemic diarrhea virus strains circulating in the regions around Hunan, China, during 2017-2018. *Archives of Virology*.

[33] Hofmann M., Wyler R. (1988). Propagation of the virus of porcine epidemic diarrhea in cell culture. *Journal of Clinical Microbiology*.

[34] Yang D., Su M., Li C. (2020). Isolation and characterization of a variant subgroup GII-a porcine epidemic diarrhea virus strain in China. *Microbial Pathogenesis*.

[35] Wu Q., Li J., Wang W. (2021). Next-generation sequencing reveals four novel viruses associated with calf diarrhea. *Viruses*.

[36] Huang C. Y., Draczkowski P., Wang Y. S. (2022). In situ structure and dynamics of an alphacoronavirus spike protein by cryo-ET and cryo-EM. *Nature Communications*.

[37] Li C., Li W., Lucio de Esesarte E. (2017b). Cell attachment domains of the porcine epidemic diarrhea virus spike protein are key targets of neutralizing antibodies. *Journal of Virology*.

[38] Zhu T., Du S., Cao D. (2019). Isolation and identification of a variant subtype G 2b porcine epidemic diarrhea virus and S gene sequence characteristic. *Infection, Genetics and Evolution*.

[39] Dong N., Fang L., Yang H. (2016). Isolation, genomic characterization, and pathogenicity of a Chinese porcine deltacoronavirus strain CHN-HN-2014. *Veterinary Microbiology*.

[40] Wen Z., Li J., Zhang Y. (2018). Genetic epidemiology of porcine epidemic diarrhoea virus circulating in China in 2012-2017 based on spike gene. *Transboundary and Emerging Diseases*.

[41] Van Diep N., Choijookhuu N., Fuke N. (2020). New tropisms of porcine epidemic diarrhoea virus (PEDV) in pigs naturally coinfected by variants bearing large deletions in the spike (S) protein and PEDVs possessing an intact S protein. *Transboundary and Emerging Diseases*.

[42] Lee S., Kim Y., Lee C. (2015). Isolation and characterization of a Korean porcine epidemic diarrhea virus strain KNU-141112. *Virus Research*.

[43] Pan Y., Tian X., Li W. (2012). Isolation and characterization of a variant porcine epidemic diarrhea virus in China. *Virology Journal*.

[44] Chen J., Liu X., Shi D. (2013). Detection and molecular diversity of spike gene of porcine epidemic diarrhea virus in China. *Viruses*.

[45] Zhang Y., Chen Y., Yuan W. (2020). Evaluation of cross-protection between G1a- and g2a-genotype porcine epidemic diarrhea viruses in suckling piglets. *Animals*.

[46] Graham R. L., Baric R. S. (2010). Recombination, reservoirs, and the modular spike: mechanisms of coronavirus cross-species transmission. *Journal of Virology*.

[47] Lu G., Wang Q., Gao G. F. (2015). Bat-to-human: spike features determining ’host jump’ of coronaviruses SARS-CoV, MERS-CoV, and beyond. *Trends in Microbiology*.

[48] Zhou P., Fan H., Lan T. (2018). Fatal swine acute diarrhoea syndrome caused by an HKU2-related coronavirus of bat origin. *Nature*.

[49] He W. T., Bollen N., Xu Y. (2022). Phylogeography reveals association between swine trade and the spread of porcine epidemic diarrhea virus in China and across the world. *Molecular Biology and Evolution*.

[50] Chiou H. Y., Huang Y. L., Deng M. C. (2017). Phylogenetic analysis of the spike (s) gene of the new variants of porcine epidemic diarrhoea virus in Taiwan. *Transboundary and Emerging Diseases*.

[51] Lee C. (2015). Porcine epidemic diarrhea virus: an emerging and re-emerging epizootic swine virus. *Virology Journal*.

