# Supplementary material for: A Porcine Epidemic Diarrhea Virus Isolated from a Sow Farm Vaccinated with CV777 Strain in Yinchuan, China: Characterization, Antigenicity, and Pathogenicity
Source: Transbound Emerg Dis. 2023 Mar 7;2023:7082352. doi: 10.1155/2023/7082352 (PMC12016728; doi:10.1155/2023/7082352)
Supplement: Supplementary Materials — Table S1 The reference PEDV strains used in this study. Table S2 Sequences and primer pair characteristics in this study. Table S3 Multiple alignments of S protein aa sequences (n = 78) in this study. [file 7082352.f1.zip › Table S1 (2).docx]

Table S1 The reference PEDV strains used in this study

| **Strain name** | **Accession No.** | **Countries** | **Collection date** | **Genotypes** |
| --- | --- | --- | --- | --- |
| USA/OK10240-6/2017 | MG334554 | USA | 2017 | GⅡb |
| OKN-1/JPN/2013 | LC063836 | Japan | 2013 | GⅡb |
| COL/Cundinamarca/2014 | KU569509 | Colombia | 2014 | GⅡb |
| USA/Colorado/2013 | KU569509 | USA | 2013 | GⅡb |
| MEX/104/2013 | KJ645708 | Mexico | 2013 | GⅡb |
| EdoMex/103/2018 | MT490315 | Mexico | 2018 | GⅡb |
| PEDV/USA/Kansas126/2015 | KU982967 | USA | 2015 | GⅡb |
| Ukraine/Poltava01/2014 | KP403954 | Ukraine | 2014 | GⅡb |
| PEDV/UG/Canada/Ontario/2014 | MZ803010 | Canada | 2014 | GⅡb |
| USA/Illinois176/2014 | KR265763 | USA | 2014 | GⅡb |
| ON-018 | KM189367 | Canada | 2014 | GⅡb |
| SF4017 | MK558089 | Philippines | 2017 | GⅡb |
| MYG-1/JPN/2014 | LC063838 | Japan | 2014 | GⅡb |
| PC273/O | MG837058 | USA | 2017 | GⅡb |
| KNU-1907 | MW560715 | South Korea | 2019 | GⅡb |
| JX2020 | MW386982 | China | 2020 | GⅡb |
| GD-XL-2019 | MN759311 | China | 2019 | GⅡb |
| GDS08 | MH726385 | China | 2011 | GⅡb |
| YN150 | MZ581326 | China | 2017 | GⅡb |
| 17GXCZ-1ORF3c | MT547180 | China | 2017 | GⅡb |
| CHSD2014 | KX791060 | China | 2014 | GⅡb |
| PEDV YZ | MK841495 | China | 2016 | GⅡb |
| NW17 | MF782686 | China | 2015 | GⅡb |
| GDS25 | MH726365 | China | 2013 | GⅡb |
| WHLL | MN037494 | China | 2018 | GⅡb |
| CN/Liaoning25/2018 | MK796238 | China | 2018 | GⅡb |
| CH/HNYY/2018 | MT090145 | China | 2018 | GⅡb |
| GDS27 | MH726380 | China | 2014 | GⅡb |
| PEDV-LNsy | KY007140 | China | 2015 | GⅡb |
| IBT-VN | MT198679 | Viet Nam | 2018 | GⅡa |
| CH_hubei_2016 | KY928065 | China | 2016 | GⅡa |
| PEDV HM | MZ342899 | China | 2017 | GⅡa |
| H11-SD2017 | MH708243 | China | 2017 | GⅡa |
| PEDV/CH/XC/2020 | OM393722 | China | 2020 | GⅡa |
| HM2017 | MK690502 | China | 2016 | GⅡa |
| ZJ/ZX2018-C10 | MK250953 | China | 2018 | GⅡa |
| CH/SCZG/2017 | MH061337 | China | 2017 | GⅡa |
| SC2021 | OL411879 | China | 2021 | GⅡa |
| PEDV HK2021 | OL762457 | China | 2021 | GⅡa |
| PEDV SD2021 | OL762459 | China | 2021 | GⅡa |
| PEDV JX2020 | OL762460 | China | 2020 | GⅡa |
| SNJ-P | MK702008 | China | 2018 | GⅡa |
| CH/SXWS/2018 | MT090146 | China | 2018 | GⅡa |
| PEDV CH/SX/2016 | MT787025 | China | 2016 | GⅡa |
| CH/JSXZ/2015 | MT625963 | China | 2015 | GⅡa |
| CH/HNAY/2015 | KR809885 | China | 2015 | GⅡa |
| PEDV SD2020 | OL762456 | China | 2020 | GⅡa |
| PEDV TRS2021 | OL762461 | China | 2021 | GⅡa |
| SHXX1902 | MN841671 | China | 2019 | GⅡa |
| HB2018 | MT166307 | China | 2018 | GⅡa |
| CH/SCZY44/2017 | MH061338 | China | 2017 | S-INDEL |
| CH/SCMY/2018 | MH061343 | China | 2018 | S-INDEL |
| CH/SCQL623/2018 | MH593152 | China | 2018 | S-INDEL |
| CH/SCCZ/2018 | MH593148 | China | 2018 | S-INDEL |
| USA/MO/2014/03293 | KM975741 | USA | 2014 | S-INDEL |
| USA/Iowa106/2013 | KJ645695 | USA | 2013 | S-INDEL |
| COL/Cauca/2015 | MK071639 | Colombia | 2015 | S-INDEL |
| OH851 | KJ399978 | USA | 2014 | S-INDEL |
| PEDV-1481-Pamplona-Tudela | MN692774 | Spain | 2014 | S-INDEL |
| L00721/GER/2014 | LM645057 | Germany | 2014 | S-INDEL |
| PEDV/Pig-wt/ESP/Calaf-1/2014 | MT602520 | Spain | 2014 | S-INDEL |
| S236 | MH593900 | Hungary | 2018 | S-INDEL |
| FR2019001 | MN056942 | France | 2019 | S-INDEL |
| 0100/4T | MZ313556 | Poland | 2017 | S-INDEL |
| SLOreBAS-1/2015 | KY019623 | Slovenia | 2015 | S-INDEL |
| 25364/2 | MZ268115 | Poland | 2015 | S-INDEL |
| SD-M | JX560761 | China | 2012 | GⅠb |
| SD2019 | MZ596343 | China | 2019 | GⅠb |
| PEDV CH/SX/2015 | MT783684 | China | 2016 | GⅠb |
| JS-2/2015 | KX534206 | China | 2015 | GⅠb |
| Attenuated DR13 | JQ023162 | South Korea | 2012 | GⅠb |
| SM98 | GU937797 | South Korea | 1998 | GⅠa |
| CV777 | AF353511 | Belgian | 1988 | GⅠa |
| Br1/87 | LT906582 | Germany | UNKNOWN | GⅠa |
| Virulent DR13 | JQ023161 | South Korea | 1999 | GⅠa |
| CH/S | JN547228 | China | 1986 | GⅠa |
| CH5 | JQ239433 | China | 2011 | GⅠa |
