# Supplementary material for: A Porcine Epidemic Diarrhea Virus Isolated from a Sow Farm Vaccinated with CV777 Strain in Yinchuan, China: Characterization, Antigenicity, and Pathogenicity
Source: Transbound Emerg Dis. 2023 Mar 7;2023:7082352. doi: 10.1155/2023/7082352 (PMC12016728; doi:10.1155/2023/7082352)
Supplement: Supplementary Materials — Table S1 The reference PEDV strains used in this study. Table S2 Sequences and primer pair characteristics in this study. Table S3 Multiple alignments of S protein aa sequences (n = 78) in this study. [file 7082352.f1.zip › Table S2 (1).docx]

Table S2 Sequences and primer pair characteristics

| Primer | Primer sequence (5′ → 3′) | Gene | Product size (bp) |
| --- | --- | --- | --- |
| P1 | CCGGAATTCATGAGGTCTTTAATTTACTTCTGGT | CV777 S1 | 2376 |
| P2 | AATACTCATACTAAAGTTGGTGGGA |  |  |
| P3 | TCCCACCAACTTTAGTATGAGTATT | CV777 S2 | 1810 |
| P4 | CCGCTCGAGTCACTGCACGTGGACCTTTTCAAAA |  |  |
| P5 | CCGGAATTCATGAAGTCTTTAACTTACTTCTGGT | CH/Yinchuan/2021 S1 | 2385 |
| P6 | AATACTCATACTAAAGTTGGTGGGA |  |  |
| P7 | TCCCACCAACTTTAGTATGAGTATT | CH/Yinchuan/2021 S2 | 1819 |
| P8 | CCGCTCGAGTCACTGCACGTGGACCTTTTCAAAA |  |  |
